# Supplementary material for: Interaural time difference sensitivity under binaural cochlear implant stimulation persists at high pulse rates up to 900 pps
Source: Sci Rep. 2023 Mar 7;13:3785. doi: 10.1038/s41598-023-30569-0 (PMC9992369; doi:10.1038/s41598-023-30569-0)
Supplement: Supplementary file 1 — Supplementary Information. [file 41598_2023_30569_MOESM1_ESM.docx]

## Supplementary Materials:

1.
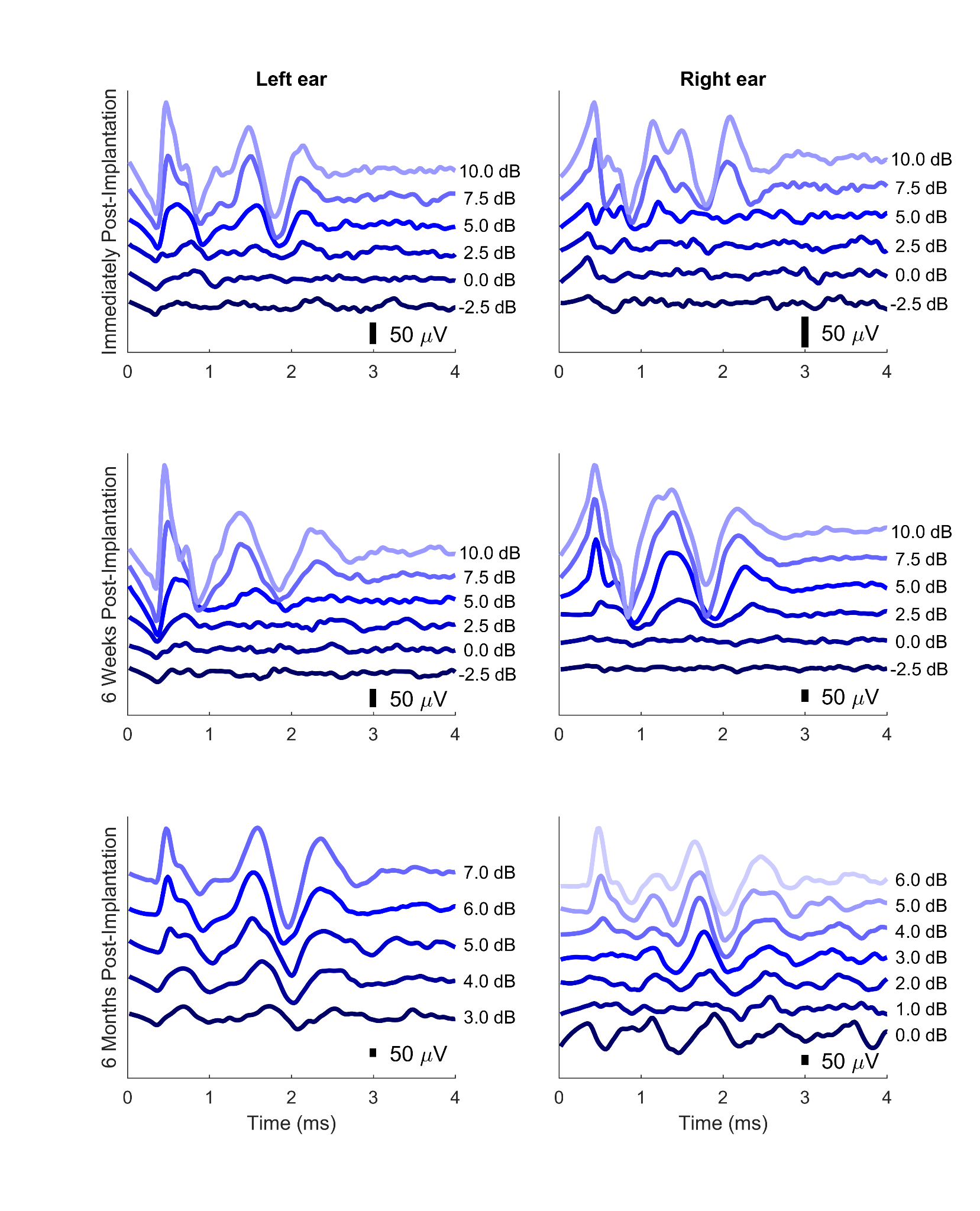


**Figure 1 - figure supplement 1 (S1):** Example eABRs showing immediately after bilateral CI implantation, 6 weeks, and 6 months after implantation thresholds for the left and right ears of a neonatally deafened rat. Scale bars are shown in each plot with reference to 50 μV. Colour represents a different SPL with dark to light colors going from softest to loudest, and 0 dB SPL corresponding to 100 µA current level. Electric artifacts have been removed using interpolation over the duration of the stimulus. For details on the stimulus and presentation, see [11].


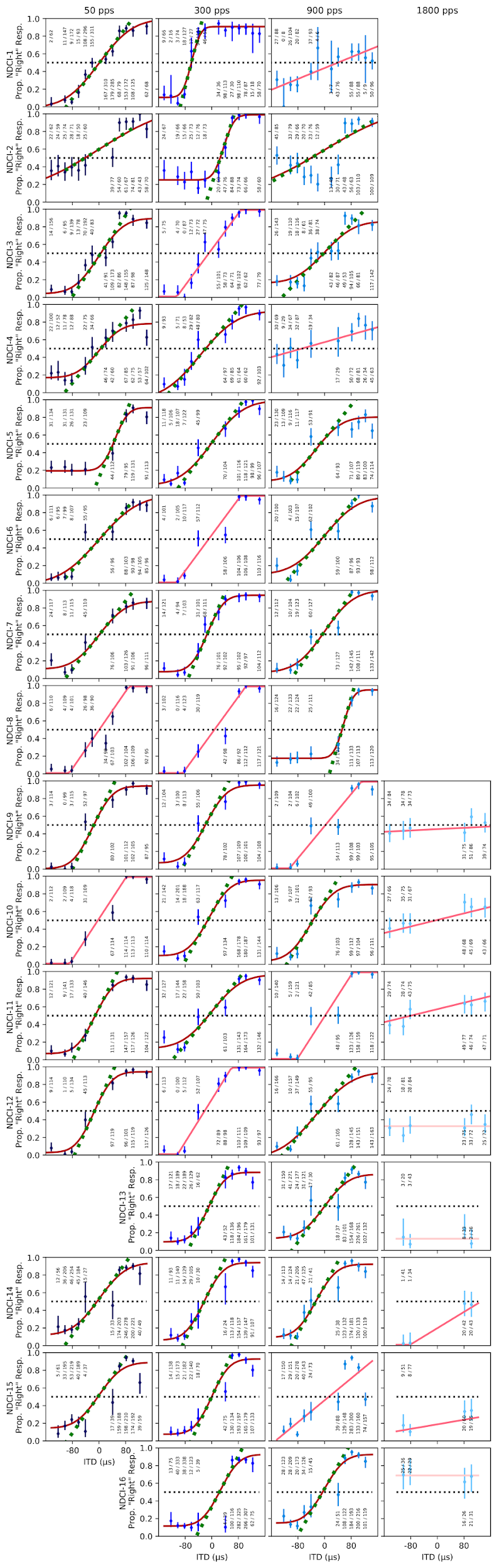
**Figure 2 - figure supplement 2 (S2):** Psychometric functions of all 16 CI rats for rectangular windowed pulse trains at each pulse rate. Each column represents a different pulse rate from 50, 300, 900 and 1800 pulses per second (pps) (left to right). Each row shows the responses for a given animal. The y-axis reflects “right” responses where “right” refers to the right hand spout (Prop. “Right” Resp.). The x-axis shows the tested interaural time difference (ITD) values from – 150 to + 150 µs. Negative ITD values indicate left leading ITDs. Annotations above or below each marker indicate the number of trials the animal chose the right hand side spout over the total number of presentations for a given ITD value. From dark to light the different shades of red indicate sigmoid, linear with bounds or null model psychometric curve fits. Green dashed lines show slopes of psychometric curves at ITD=0 µs. Slopes serve to quantify the behavioral sensitivity of the animal to ITD.


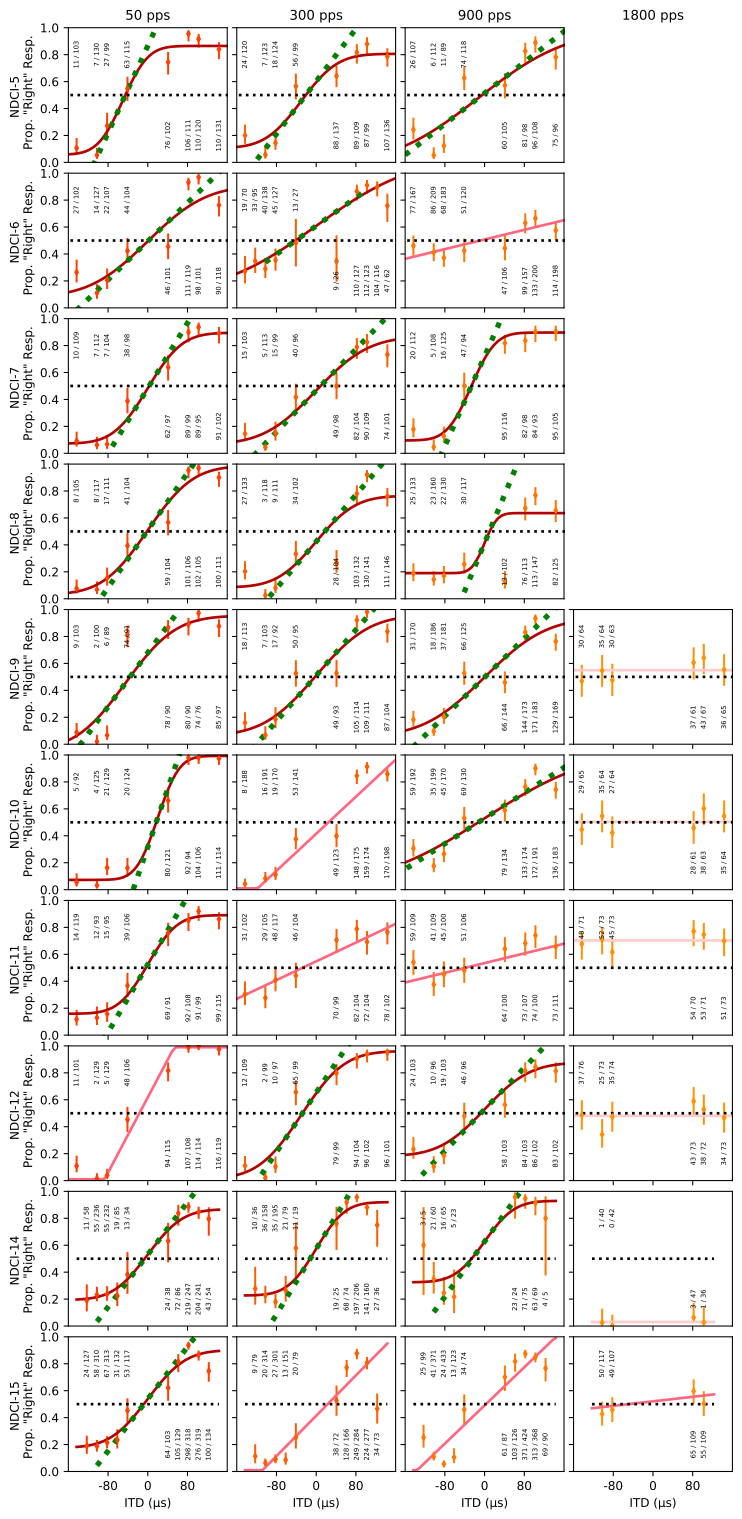


**Figure 3 - figure supplement 3 (S3):** Psychometrics of 10 CI rats for Hanning windowed pulse trains with varying ITDs as in Figure S2**.** Details are as for S2.
